# Development of Nanosuspension Formulations Compatible with Inkjet Printing for the Convenient and Precise Dispensing of Poorly Soluble Drugs

**DOI:** 10.3390/pharmaceutics14020449

**Published:** 2022-02-19

**Authors:** Dennis H. Leung

**Affiliations:** Small Molecule Pharmaceutical Sciences, Genentech, Inc., 1 DNA Way, South San Francisco, CA 94080, USA; leung.dennis@gene.com

**Keywords:** nanosuspension, formulation, inkjet, printing, personalized medicine, thin film

## Abstract

The pharmaceutical industry has been challenged by the increasing number of poorly soluble drug candidates, resulting in significant issues with obtaining sufficient absorption and bioavailability, risk of exposure variability, and difficulties in achieving a safe therapeutic index. Additionally, the rapid and precise dispensing of specific drug dosages is an important aspect that can enable personalized medicines for the patient. Herein, we report on the development of inkjet printing as a method for delivering precise quantities of poorly soluble drug molecules using commercially available equipment. Despite challenges due to low solubility making it difficult to prepare liquid solutions, stable suspensions of drug nanoparticles with the appropriate viscosity were successfully printed and dispensed onto a thin film suitable for delivery. The drug nanoparticles remained intact and could be reconstituted after printing, demonstrating that they remained stable and retained their advantageous particle size. This demonstrates that inkjet printing can be a practical and convenient approach for dispensing poorly soluble drug molecules when formulated as nanosuspensions.

## 1. Introduction

Traditionally, the development of drug products has often taken a “one-size-fits-all” approach with respect to dosage and image, most commonly exemplified by standard tablet sizes. This requires the development of a suitable bulk manufacturing approach and supply chain with the appropriate quality control in order to subsequently deliver the drug product at those specific doses to the target patient population. However, increasing understanding of disease pathology and its relationship with genetics, as well as an increased amount of statistical data that can be obtained for each patient, has driven significant interest in personalized medicine approaches [1,2]. Furthermore, current manufacturing approaches for solid dosage forms, such as tablets, face significant challenges with consistency and content uniformity for low dosages, which are particularly important for highly potent drugs as well as for pediatric indications. As a result, the ability to manufacture precise dosage forms on-demand which can be tailored for an individual patient while handling low quantities of drugs has significant value. In addition to providing a personalized and more efficacious therapy for the patient, this could potentially minimize the logistics involved in current drug product manufacture.

One intriguing response to this challenge is leveraging on-demand printing technology. Techniques such as 3D printing have been studied intensely in recent years as an approach to manufacturing solid dosage drug product formulations [3,4,5,6,7]. As a complementary approach, inkjet printing has emerged as a method for preparing drug formulations [8,9]. Inkjet printing is well-established and consumer inkjet printers are relatively inexpensive and commonly available. Inkjet printing has been designed and optimized for the precise control and dispensing of tiny volumes of liquid droplets, potentially making it an ideal platform for use with liquid drug formulations. Thus, the ability to take advantage of the current inkjet printing infrastructure could enable rapid deployment and implementation of this approach.

In recent years, there has been some initial work at using inkjet printing as a technique for dispensing and manufacturing personalized dosage forms [8,9,10,11,12,13,14,15,16,17,18,19,20,21,22]. In addition to its ubiquitous nature, inkjet printing has several potential advantages: (1) precise dispensing of ultra-low quantities of the drug, ideal for highly potent molecules; (2) personalized dosage forms tailored to individual patients; (3) on-demand printing to enable immediate consumption, minimizing long term stability risks; (4) production of thin-film formulations amenable for rapid disintegration and release in a variety of delivery routes.

While inkjet printing has many potential advantages as a drug manufacture technology, much of the work in this area has been limited to the use of soluble drug compounds dissolved in solution acting as the “ink”. For example, Gaisford and coworkers reported the ability to dispense salbutamol sulfate using inkjet printing [15]. In another example, Genina et al. demonstrated inkjet printing of solutions of loperamide hydrochloride and caffeine [16]. These approaches worked well, as the model drug compounds were highly soluble in the aqueous solutions used in the printing process. However, a significant fraction of new drug molecules tend to be poorly soluble in aqueous media, making these approaches challenging [23]. An initial report by Pardeike et al. demonstrated that small suspensions of folic acid could be compatible with a microdosing dispenser [22]. Thus, a method for enabling the inkjet printing of poorly soluble molecules in safe aqueous solutions would be highly desirable.

This report demonstrates a practical approach for the development of stable nanosuspensions formulations as a general “ink” for insoluble drug molecules amenable for precise dispensing with inkjet printing using commonly available off-the-shelf equipment without the need for extensive modification. Nanosuspensions are formulations of colloidal drug nanoparticles that can significantly enhance the absorption of poorly soluble molecules. Importantly, the drug nanoparticles remain stable during and after the inkjet printing process. Reconstitution of the printed film using aqueous media restores the suspended nanoparticles, indicating that the drug formulation retains its beneficial qualities for absorption in vivo after printing.

## 2. Materials and Methods

### 2.1. Materials

Reagents and materials were obtained from commercial sources and used as received unless otherwise noted. Loadings and concentrations are reported as weight percent (wt. %) unless otherwise noted.

### 2.2. Manufacture of Naproxen Nanosuspension Using Resonant Acoustic Mixing

Naproxen (Sigma Aldrich, St. Louis, MO, USA), PVP K29-32 (Spectrum Chemical, Gardena, CA, USA), and sodium dodecyl sulfate (SDS, Spectrum Chemical, Gardena, CA, USA) were used as received commercially. The resonant acoustic milling approach reported previously was used [24,25]. A 20-mL glass scintillation vial was charged with 45.6 g of YTZ Tosoh zirconia milling beads (Tosoh USA Inc., Grove City, OH, USA). Naproxen solid (0.876 g) was weighed into the vial. In a separate 20-mL vial, water (2.628 mL) and a stock solution of 5% PVP K29-32 polymer (4.380 mL) and 1% SDS (0.876 mL) were mixed. The aqueous solutions were then transferred to the vial containing naproxen to form a slurry at a naproxen:PVP K29-32:SDS ratio of 1:0.25:0.01 and an overall drug concentration of 100 mg/mL. The vial was sealed with a Teflon cap and then placed on a Resodyn LabRam resonant acoustic mixer (Resodyn Acoustic Mixers, Butte, MT, USA) to mill at an intensity of 40 Gs for 2 h.

The resulting liquid nanosuspension sample was removed from the milling beads by extraction with a syringe equipped with a 26-gauge needle, giving an average yield of approximately 7 g (80%). The nanosuspension at 100 mg/mL drug concentration was diluted with the appropriate volume of water to afford formulation samples at 10 mg/mL and 1 mg/mL drug concentration. In some cases, glycerol (Sigma Aldrich, St. Louis, MO, USA) was added as a viscosity-modifying agent.

### 2.3. Particle Size Analysis Using Dynamic Light Scattering

The particle size distribution of the naproxen formulations as prepared was determined. A 10 µL aliquot of the nanosuspension sample was taken and diluted in 90 µL of water for analysis using a Wyatt DynaPro^TM^ Plate Reader II dynamic light scattering instrument (Wyatt Technology Corporation, Santa Barbara, CA, USA). A 40 µL aliquot of the diluted suspension was dispensed into a Corning^®^ low volume black polystyrene 384-well plate for analysis (Corning Life Sciences, Tewksbury, MA, USA). The particle size of each sample was reported as an average of 10 acquisitions with an acquisition time of 3 sec at 25 °C. Autocorrelation curves were fit using either the cumulants or regularization method, and the average particle diameter D50 and D90 were obtained and reported. The normalized polydispersity (%Pd) was calculated as the polydispersity divided by the estimated hydrodynamic radius from the cumulants fit of the autocorrelation function and multiplied by 100.

### 2.4. Viscosity Measurements

The viscosity of the naproxen formulations was measured using a Cambridge ViscoLab 3000 viscometer (Cambridge Viscosity, Inc., Boston, MA, USA). A recirculating water bath was used to maintain a temperature of 25 °C at the sample chamber. An aliquot of the formulation sample (0.5 mL) was added to the sample chamber, and the viscosity was recorded after the measurements had stabilized to <0.1% variation.

### 2.5. Surface Tension Measurements

The surface tension of the naproxen formulations was measured using an Attension Theta optical tensiometer (Biolin Scientific, Gothenburg, Sweden). An aliquot of the formulation (0.5 mL) was added to the sample stage and the contact angle of the droplet was recorded.

### 2.6. Inkjet Printing

A commercial Canon PIXMA iP7220 Wireless Inkjet Photo Printer (Canon USA Inc., Huntington, NY, USA) equipped with a standard thermal inkjet printhead was used for these studies. Generic third-party refillable ink cartridges were used as received and loaded with the liquid formulations for printing (Amazon.com, Inc., Seattle, WA, USA). Adobe Photoshop (Adobe, San Jose, CA, USA) was used to create a simple printing pattern consisting of a circle with a diameter of 100 pixels and with a resolution of 72 dots per inch (dpi) using an ink color from a single cartridge source (e.g., cyan). Commercial 8.5” × 11” acetate transparency film was used as the printing substrate. The printed output was allowed to air dry for several hours before analysis. Between printing runs, the inkjet printhead was carefully washed with deionized water and acetone to remove any residual formulation.

### 2.7. HPLC Analysis

A suitable method for naproxen concentration determination was developed internally and validated using standards of known concentration. Drug concentration analyses were performed on an Agilent 1100 system (Agilent Technologies Inc., Santa Clara, CA, USA) equipped with a quaternary pump, a photodiode array UV-Vis detector, and a Waters XTerra RP18 column (3.5 μm particle size, 50 × 4.6 mm dimension). The mobile phases consisted of 0.05% trifluoroacetic acid in water and in acetonitrile. Gradient elution was utilized with the acetonitrile mobile phase increasing from 2 to 65% over 6.5 min at a flow rate of 1.25 mL/min. The column was heated at 30 °C and the detection was carried out at 254 nm.

### 2.8. Microscopy Analysis

A Leica DM6000 M automated research microscope (Leica Microsystems Inc., Buffalo Grove, IL, USA) equipped with a Lumenera microscopy camera (Teledyne Lumenera, Ottawa, ON, Canada) was used for optical microscopy analysis. Commercially available acetate transparencies used for inkjet printing, and the nanosuspensions formulations were directly analyzed by brightfield transmitted light microscopy at various magnification scales. For the microdissolution and redispersability studies, a drop of water was added to the transparent film and the wetting and dissolution of the inkjet-printed nanosuspensions formulation was observed in real-time.

## 3. Results and Discussion

Inkjet printing has become common technology and a large number of consumer-grade inkjet printers are commercially available. This technology relies on the precise control and deposition of fine ink droplets onto a thin-film substrate, such as paper or plastic polymeric sheets. In many systems, these ink droplets are controlled via a fixed printhead, which typically consists of thousands of tiny nozzles for the rapid printing of various color inks using piezoelectric or thermal technologies. This process has been used for a wide variety of emerging applications, including organic and inorganic thin-film deposition, electronics, and devices, among others [26,27]. Although these shear and temperature conditions are relatively high, the impact on the liquid is relatively minor due to its transient nature and previous reports have shown that mammalian cells have survived this process intact [28]. A separate cartridge acts as a reservoir for the ink liquid and is typically mounted above the printhead. In recent years, a plethora of third-party refillable ink cartridges have become commonly available. These ink cartridges are compatible with many consumer-grade inkjet printers and allow the user to load them with generic inks or, in this case, other liquids of interest.

As can be seen, over the years, inkjet printing has been optimized for extremely fine control over liquid dispensing and has become a commoditized technology. This has potential attractive advantages for the dispensing of liquid pharmaceutical formulations. In fact, there have been several reports in recent years of taking advantage of inkjet printing for preparing thin-film formulations [8,9,10,11,12,13,14,15,16,17,18,19,20,21,22]. However, these studies have typically relied upon soluble drug solutions. Nevertheless, many new drug candidates often exhibit poor solubility and solutions cannot be prepared without the use of extensive and potentially toxic organic co-solvents [23]. In addition, in many cases, solution formulations containing large amounts of co-solvents readily precipitate upon contact with aqueous media, such as biological fluids, limiting their bioperformance in vivo. Removal of the organic solvents after deposition results in drug solids that remain poorly soluble, with a high risk of limited absorption and bioavailability. In contrast, aqueous formulations of these compounds often result in relatively large suspensions of the insoluble drug, with typical particle sizes ranging from 50–500 µm. The nozzles on standard inkjet printheads are commonly in the range of 20–120 µm [12], making the dispensing of conventional drug suspensions challenging due to the high risk of clogging. As a result, this has become a significant limitation to the applicability of inkjet printing for a broader set of drug compounds.

However, nanosuspensions have emerged as one enabling formulation approach for the formulation of poorly soluble drug candidates [29,30,31,32,33,34,35,36,37,38,39,40,41,42,43,44]. Nanosuspensions are colloidal suspensions of drug nanocrystals with sizes generally between 100–400 nm in diameter. Resonant acoustic milling technology has recently been developed as a small-scale, high throughput method for rapidly identifying suitable formulation compositions to form stable drug nanosuspensions, making this a relatively general approach for many poorly soluble compounds [24,25]. Due to the small size of the suspended particles, nanosuspensions exhibit a rapid dissolution rate and absorption in vivo, as well as maintaining low viscosity fluid-like behavior even at relatively high concentrations [32,36,45]. These favorable properties make nanosuspension formulations attractive as potential “ink” for poorly soluble compounds that would be compatible with inkjet printing.

**Figure 1:**
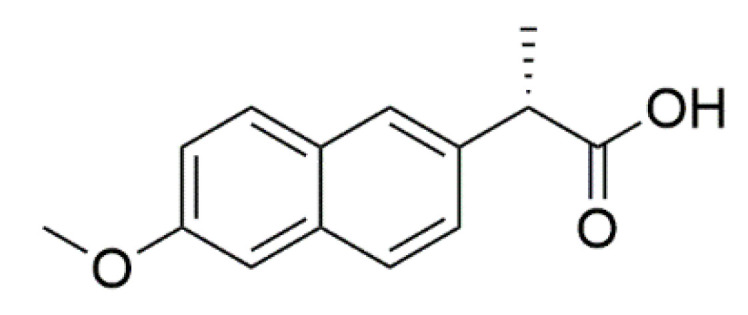
Naproxen model compound used in nanosuspensions formulations.

**Figure 2:**
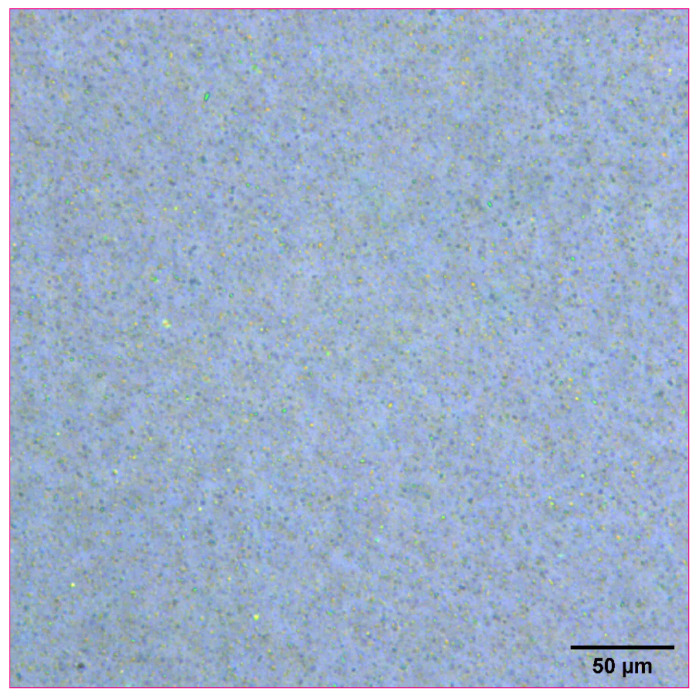
Optical microscope image of naproxen nanosuspension at a concentration of 100 mg/mL.

Naproxen was selected as a representative model compound for these studies (Figure 1). Naproxen is a non-steroidal anti-inflammatory drug (NSAID) that is practically insoluble in water that would benefit from rapid absorption in vivo. For these studies, a high concentration nanosuspensions of naproxen was used as a model formulation. Naproxen starting material was nanomilled at a concentration of 100 mg/mL using resonant acoustic mixing in the presence of small amounts of PVP K29-32 and SDS stabilizing excipients to form nanoparticles of approximately 111 nm in radius [24,25]. The resulting nanosuspension appears as a white, opaque “solution” with well-dispersed suspended nanoparticles only visible under high magnification with optical microscopy (Figure 2).

**Figure 3:**
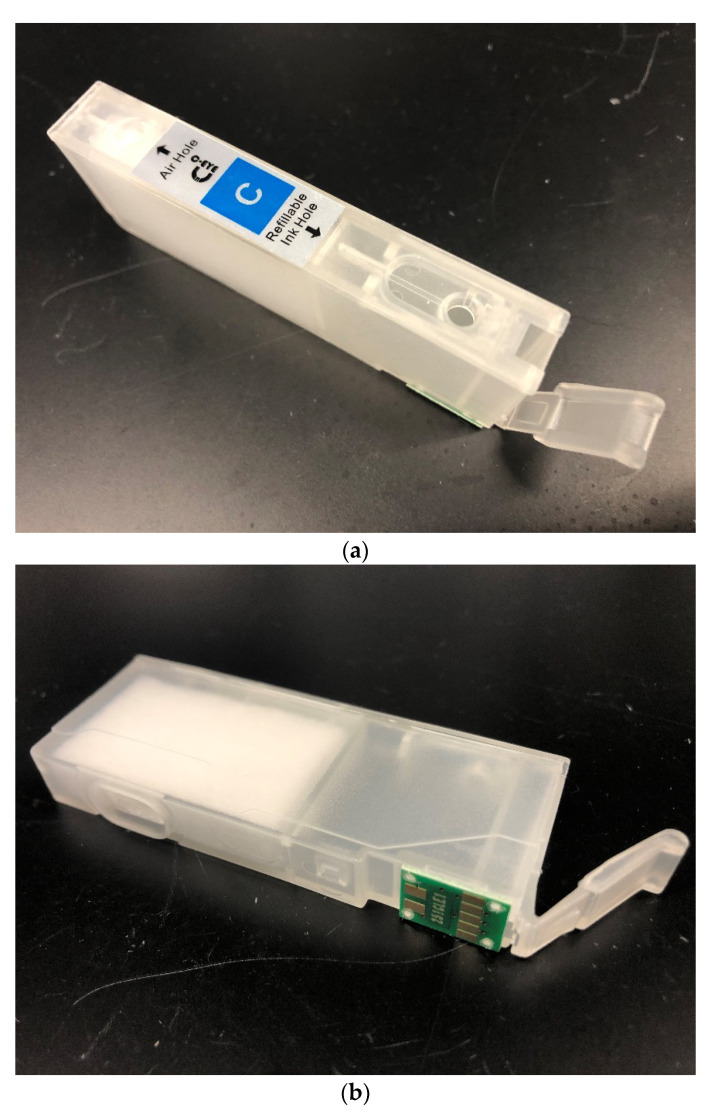
(**a**) Top view of refillable inkjet cartridge displaying the refillable ink hole. (**b**) Side and bottom view of refillable inkjet cartridge showing the internal ink reservoir and dispensing hole.

To test the compatibility of the nanosuspension ink for printing, a commercial inkjet printer, the Canon PIXMA iP7220, was used for these studies. This printer is equipped with a Canon QY6-0082 thermal inkjet printhead for drop-on-demand printing use with five individual ink tanks. In order to ensure that the printhead remained unclogged during these experiments due to the presence of a residual drug, the inkjet printhead was carefully washed with deionized water and acetone to remove any residual formulation. Commercially available third-party refillable ink cartridges were used that were compatible with loading various liquid formulations easily (Figure 3). Liquid ink is introduced to the cartridge via an aperture at the top, which then fills the internal reservoir. The liquid can then be dispensed through an aperture at the bottom of the cartridge, where it then enters the inkjet printhead for the final dispense. The refillable ink cartridge can be washed with water or organic solvents and reused for further studies. This provides a convenient system for loading and testing a variety of liquid formulations.

The liquid naproxen nanosuspensions were then added to the refillable inkjet cartridges. Although the nanosuspensions consisted of fine particles not expected to clog the inkjet nozzles, the importance of other physical properties, such as the viscosity of the liquid to enable printing, became readily apparent [46,47,48]. Commercial inks used for inkjet printing typically have a proprietary formulation composition but tend to consist of an aqueous-based liquid with viscosities between ~1–5 cP [49]. This range appears to be optimized for the printing process, as liquids that are too viscous are unable to be expelled from the inkjet nozzle while liquids that have low viscosities freely flow through the nozzle in an uncontrolled manner. Similarly, viscosity was also a critical factor for use of the refillable cartridges used here. Liquids of relatively low viscosity were not retained within the cartridge and tended to flow freely out of the dispensing aperture at the bottom of the cartridge.

In order to identify the optimal viscosity values for these formulations, a series of naproxen nanosuspensions were prepared at various drug concentrations. The intrinsic viscosities were then measured using a Cambridge ViscoLab 3000 viscometer (Table 1). The formulations were then loaded into the refillable ink cartridge and tested. The viscosity of these formulations is dependent on drug concentration but can also be tuned through the addition of viscosity-modifying excipients, such as glycerol. The nanosuspensions at 100 mg/mL naproxen concentration had a viscosity of 2.146 cP, which was high enough to enable the formulation to be maintained within the cartridge while also facilitating effective printing. However, more dilute nanosuspensions formulations at 10 and 1 mg/mL exhibited a lower viscosity. These formulations were not effectively retained within the ink cartridge and could not be printed.

In order to overcome this limitation and allow a wider range of drug concentrations to be printed, the addition of glycerol as a viscosity-enhancing excipient was investigated. Glycerol is a highly viscous organic cosolvent that is miscible with water. However, it was critical to determine the effect that the addition of glycerol might have on the stability of the nanosuspension. In particular, increased solubilization of the drug nanoparticles due to changes in the solvent media can result in Ostwald ripening and aggregation.

The particle size stability of the naproxen nanosuspensions was tested with small additions of glycerol and is shown in Table 2. The addition of 10 and 20% glycerol to the nanosuspensions resulted in a slight increase in particle size, suggesting that particle growth and aggregation were potential risks. Based on the viscosity measurements, 10% glycerol appeared to be a good balance, providing formulations of the optimal viscosity for facilitating printing as well as maintaining good particle size stability for further testing.

In addition to viscosity, surface tension can also play a key role in droplet formation during the inkjet printing process [46,47,48]. The surface tension of the nanosuspension formulations was measured using an Attension Theta optical tensiometer. Since the formulations included surface-active excipients such as SDS, the surface tension was concentration-dependent, ranging from 34.9 mN/m for the most concentrated formulation at 100 mg/mL for the least concentrated formulation at 1 mg/mL (Table 1). As can be seen, once the viscosity of the formulations had been modified using the non-surface-active agent glycerol, the 10 mg/mL nanosuspension formulations were capable of being printed, suggesting that the inkjet printing process was compatible with a wide range of surface tensions.

**Figure 4:**
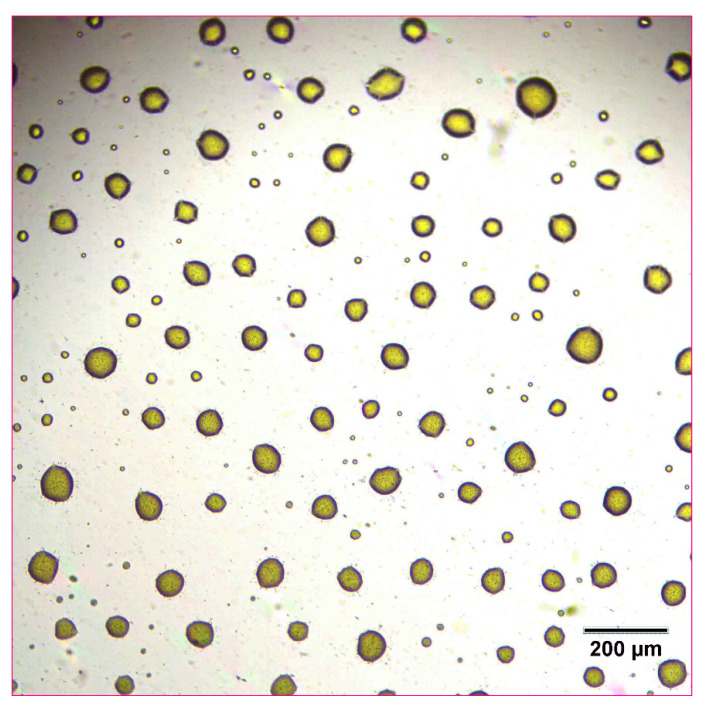
Optical microscope image of 10 mg/mL concentration naproxen nanosuspension after inkjet printing onto a film transparency at 100× magnification.

**Figure 5:**
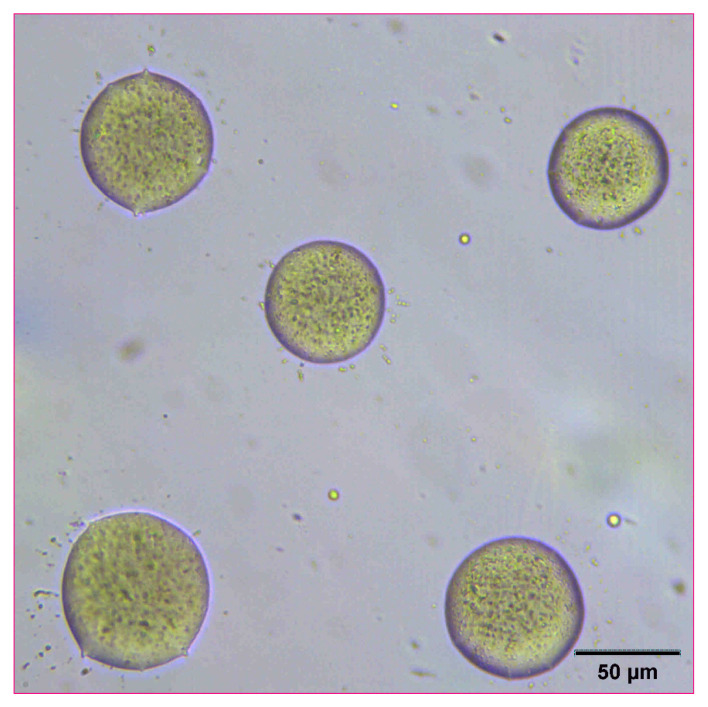
Optical microscope image of 10 mg/mL concentration naproxen nanosuspension after inkjet printing onto a film transparency at 500× magnification.

The 10 mg/mL naproxen nanosuspensions +10% glycerol formulation was used for further inkjet printing studies. A solid circular pattern consisting of a single ink color (e.g., cyan) was designed and used for printing tests. The refillable ink cartridge was loaded into the printer and the pattern was printed onto a commercially available acetate film transparency. Upon examination with optical microscopy, the circular pattern was resolved into individual ink droplets of consistent size containing the suspended nanoparticles dried on the film surface (Figure 4 and Figure 5). The droplets were distributed evenly across the film in a consistent and even manner, corresponding to the resolution of the printed image (72 dpi). At a higher magnification of 500x, the nanoparticles in the individual ink droplets could begin to be resolved. The behavior of the droplets, including their shape, is likely controlled by the surface tension of the liquid formulation and may be a parameter for further optimization.

The amount of naproxen deposited via inkjet printing was then analyzed. The printed patterns were washed and fully dissolved with 1:1 acetonitrile:water. The resulting solutions were then analyzed by HPLC with drug concentrations compared to standards of known concentration. Analysis of multiple printed patterns (*n* = 3) indicated that the deposition of microgram quantities of the drug onto the transparency film was highly reproducible with low variability (Table 3). The amount of deposited drug is also consistent with the estimated droplet size for this printhead. For the naproxen nanosuspension formulation with a drug concentration of 100 mg/mL, given the measured deposition of 13.19 µg/cm^2^ and the target print resolution of 72 dpi in the pattern, a droplet size of 16.6 picoliters was calculated. This is well within the expected range for droplet sizes dispensed by similar inkjet nozzles. This also indicates that there was no major disruption of droplet formation nor significant loss or degradation of the drug formulation during the printing process despite the range of viscosity and surface tension values, which is consistent with the HPLC analytical results.

These results demonstrate that the inkjet printing process is a precise method for controlling droplet deposition, with no significant issues occurring despite replacing traditional printer ink with a drug nanosuspension “ink” and enabling the manufacture of a consistent dosage form with good content uniformity. Moreover, the amount of drug deposited was directly proportional to the initial concentration of the nanosuspension ink. Reducing the initial concentration from 100 mg/mL to 10 mg/mL resulted in a 10-fold reduction in the amount of naproxen deposited onto the transparency film. In addition, HPLC analysis showed no presence of degradants or other impurities, suggesting that the drug remained stable throughout the inkjet printing process, despite the flash heating required for deposition.

**Figure 6:**
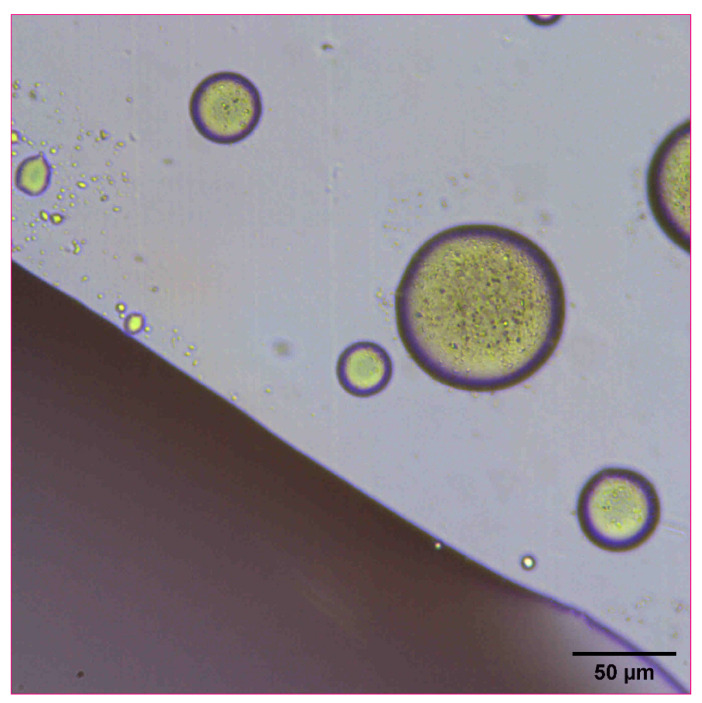
Optical microscope image of 10 mg/mL concentration naproxen nanosuspension after inkjet printing onto a film transparency. A drop of water was added to observe the redispersion and reconstitution of the dried nanosuspension droplets.

**Figure 7:**
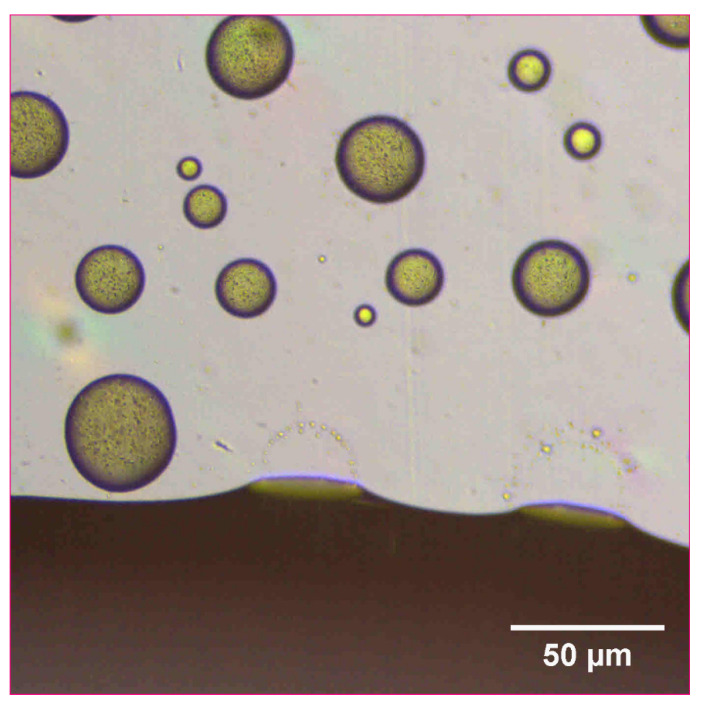
Optical microscope image of 10 mg/mL concentration naproxen nanosuspension after inkjet printing onto a film transparency. A drop of water was added to observe the redispersion of the dried nanosuspension droplets.

**Figure 8:**
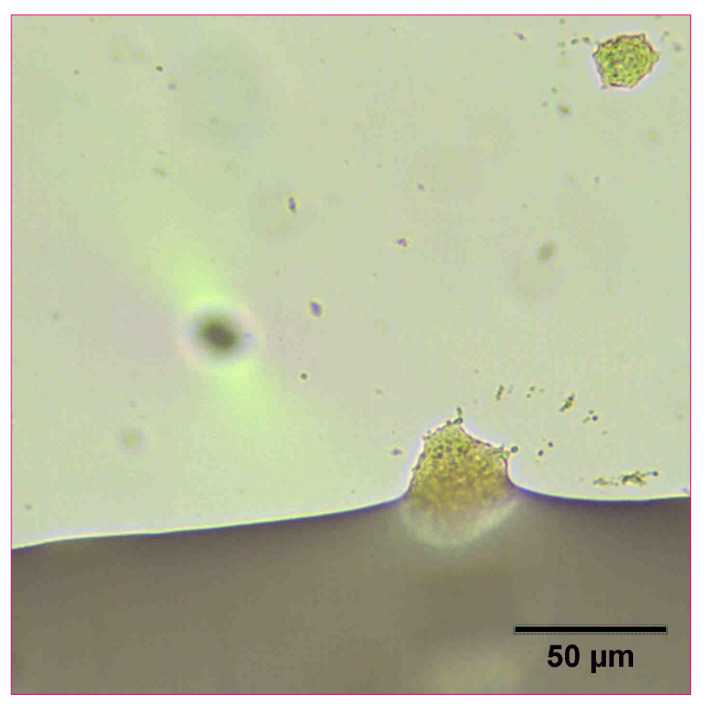
Optical microscope image of 10 mg/mL concentration naproxen nanosuspension after inkjet printing onto a film transparency. A drop of water was added to observe the redispersion of the dried nanosuspension droplets.

**Figure 9:**
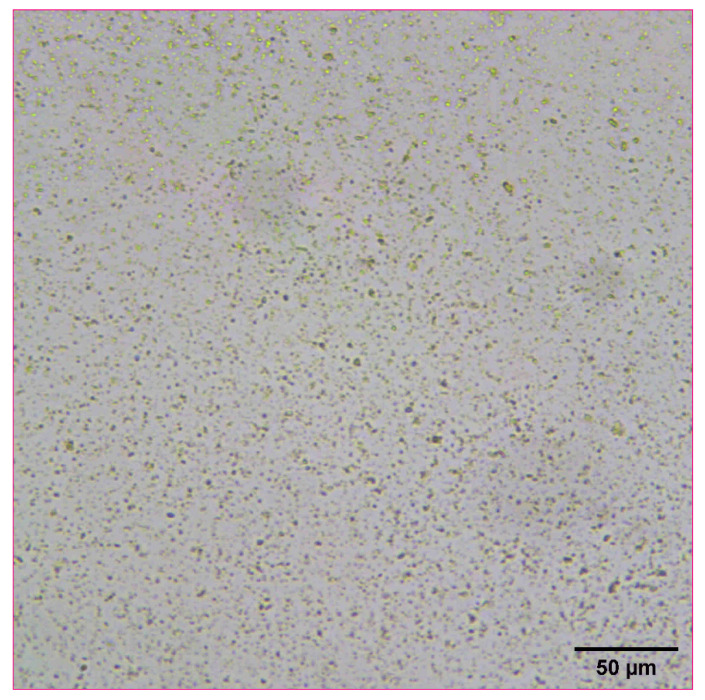
Optical microscope image of 10 mg/mL concentration naproxen nanosuspension after reconstitution with water.

The reconstitution and dissolution of the printed nanosuspensions after drying were then investigated. A drop of water was placed onto the transparency. The wetting and redispersion of the nanosuspension droplets were observed in real-time by monitoring the advancing water line with optical microscopy (Figure 6, Figure 7 and Figure 8). Upon exposure to water, the dried nanosuspension droplets were immediately hydrated, releasing the drug nanoparticles into solution. The resulting reconstituted nanosuspension was recovered and analyzed by optical microscopy, showing a homogeneous, well-dispersed suspension with no significant aggregation (Figure 9). DLS analysis of the reconstituted sample showed particles consisting of 114 nm in diameter, showing no significant aggregation after drying and redispersion, confirming that the stability of the nanosuspension during this process was maintained.

## 4. Conclusions

The ability to precisely dispense specific quantities of a drug would be highly valuable for enabling a personalized medicine approach for patients. Inkjet technology has been developed as a highly consistent approach for depositing fine liquid particles. Consumer-grade inkjet printers are commonly available, and this study demonstrates that common off-the-shelf equipment can be used for pharmaceutical applications. The results reported here indicate that precise and reproducible quantities of a poorly soluble naproxen drug nanosuspension can be inkjet-printed onto a thin film across a wide range of concentrations with no impact on particle size or chemical and physical stability during the process. Importantly, after drying, the drug nanoparticles remain stable and can successfully be reconstituted and redispersed by the addition of water, retaining the advantages of their small particle size.

This compatibility of using nanosuspension formulations as a suitable “ink” enables inkjet printing to be used as a general approach for poorly soluble drug molecules, significantly expanding its utility. The resulting material consists of a thin film of ultra-low microgram quantities of drug nanoparticles, making this suitable for the preparation of drug products with low dosages that are challenging for traditional solid dosage forms and has implications for products for highly potent molecules or pediatric formulations. In addition, the final thin-film drug product image potentially enables rapid dissolution and release of the drug, enabling fast absorption and delivery. Inkjet printing also allows for the custom tailoring of individual dosage forms on-demand through control of the initial drug concentration in the formulation as well as adjustment of the printing pattern. Moreover, the results described here demonstrate that this approach can be undertaken with common commercially available off-the-shelf equipment with little custom modification. One can envision the potential for taking advantage of the existing inkjet printing infrastructure by providing ink cartridges containing a nanosuspension formulation, enabling a general approach for personalized medicine. Further studies are underway to characterize and control the behavior of the nanoparticle thin-film layer, as well as to test these drug products in vivo.

## Figures and Tables

**Table 1:** Amenability of nanosuspensions formulations of varying concentration and viscosity for inkjet printing.

Nanosuspension Concentration	Viscosity (cP)	Surface Tension (mN/m)	Printable?
100 mg/mL	2.146	34.9	Yes
10 mg/mL	1.022	49.8	N
1 mg/mL	0.958	59.1	N
10 mg/mL + 10% glycerol	1.548	---*	Yes

* Not measured since glycerol is not considered surface-active and would have a negligible effect on surface tension.

**Table 2:** Effect of addition of glycerol as a viscosity enhancer on the particle size of naproxen nanosuspensions formulations.

Nanosuspension Composition	Radius (nm)
10 mg/mL naproxen	111
10 mg/mL naproxen + 10% glycerol	134
10 mg/mL naproxen + 20% glycerol	167

**Table 3:** Deposition analysis of printed samples of naproxen nanosuspensions at 100 and 10 mg/mL concentration.

Ink Formulation Concentration	Deposition (μg/cm^2^)
100 mg/mL naproxen	13.19 ± 0.28
10 mg/mL naproxen + 10% glycerol	1.22 ± 0.04

## Data Availability

The data presented in this study are available upon request from the corresponding author.
